# A deep insight into CRISPR/Cas9 application in CAR-T cell-based tumor immunotherapies

**DOI:** 10.1186/s13287-021-02510-7

**Published:** 2021-07-28

**Authors:** Ehsan Razeghian, Mahyuddin K. M. Nasution, Heshu Sulaiman Rahman, Zhanna R. Gardanova, Walid Kamal Abdelbasset, Surendar Aravindhan, Dmitry O. Bokov, Wanich Suksatan, Pooria Nakhaei, Siavash Shariatzadeh, Faroogh Marofi, Mahboubeh Yazdanifar, Somayeh Shamlou, Roza Motavalli, Farhad Motavalli Khiavi

**Affiliations:** 1 Human Genetics Division, Medical Biotechnology Department, National Institute of Genetics Engineering and Biotechnology (NIGEB), Tehran, Iran; 2 DS & CI Research Group, Universitas Sumatera Utara, Medan, Indonesia; 3 College of Medicine, University of Sulaimani, Sulaymaniyah, Iraq; 4 Department of Medical Laboratory Sciences, Komar University of Science and Technology, Sulaymaniyah, Iraq; 5 Department of Psychotherapy, Pirogov Russian National Research Medical University, 1 Ostrovityanova St, 117997 Moscow, Russia; 6 Department of Health and Rehabilitation Sciences, College of Applied Medical Sciences, Prince Sattam bin Abdulaziz University, Al Kharj, Saudi Arabia; 7 Department of Physical Therapy, Kasr Al-Aini Hospital, Cairo University, Giza, Egypt; 8 Department of Pharmacology, Saveetha Dental College and Hospital, Saveetha Institute of Medical and Technical Sciences, Chennai, India; 9 Institute of Pharmacy, Sechenov First Moscow State Medical University, 8 Trubetskaya St., bldg. 2, Moscow, 119991 Russian Federation; 10 Laboratory of Food Chemistry, Federal Research Center of Nutrition, Biotechnology and Food Safety, 2/14 Ustyinsky pr, Moscow, 109240 Russian Federation; 11 Faculty of Nursing, HRH Princess Chulabhorn College of Medical Science, Chulabhorn Royal Academy, Bangkok, 10210 Thailand; 12 School of Medicine, Tehran University of Medical Sciences, Tehran, Iran; 13 Department of Pharmacology, School of Medicine, Shahid Beheshti University of Medical Sciences, Tehran, Iran; 14 Immunology Research Center (IRC), Tabriz University of Medical Sciences, Tabriz, Iran; 15 Stem Cell Transplantation and Regenerative Medicine, Department of Pediatrics, Stanford University School of Medicine, Palo Alto, CA USA; 16 Department of Applied Cell Sciences, School of Advanced Technologies in Medicine, Tehran University of Medical Sciences, Tehran, Iran; 17 Stem Cell Research Center, Tabriz University of Medical Sciences, Tabriz, Iran; 18 Department of Virology, Pasteur Institute of Iran, Tehran, Iran

**Keywords:** CRISPR-Cas9, CAR-T cell, Universal CAR-T cell, Genome editing technologies, Immune checkpoints

## Abstract

To date, two chimeric antigen receptors (CAR)-T cell products from autologous T cells have been approved by The United States Food and Drug Administration (FDA). The case-by-case autologous T cell generation setting is largely considered as a pivotal restraining cause for its large-scale clinical use because of the costly and prolonged manufacturing procedure. Further, activated CAR-T cells mainly express immune checkpoint molecules, including CTLA4, PD1, LAG3, abrogating CAR-T anti-tumor activity. In addition, CAR-T cell therapy potently results in some toxicity, such as cytokine releases syndrome (CRS). Therefore, the development of the universal allogeneic T cells with higher anti-tumor effects is of paramount importance. Thus, genome-editing technologies, in particular, clustered regularly interspaced short palindromic repeat (CRISPR)-Cas9 are currently being used to establish “off-the-shelf” CAR-T cells with robust resistance to immune cell-suppressive molecules. In fact, that simultaneous ablation of PD-1, T cell receptor alpha constant (TRAC or TCR), and also β-2 microglobulin (B2M) by CRISPR-Cas9 technique can support the manufacture of universal CAR-T cells with robust resistance to PD-L1. . Indeed, the ablation of β2M or TARC can severely hinder swift elimination of allogeneic T cells those express foreign HLA-I molecules, and thereby enables the generation of CAR-T cells from allogeneic healthy donors T cells with higher persistence in vivo. Herein, we will deliver a brief overview of the CAR-T cell application in the context of tumor immunotherapy. More importantly, we will discuss recent finding concerning the application of genome editing technologies for preparing universal CAR-T cells or cells that can effectively counter tumor escape, with a special focus on CRISPR-Cas9 technology.

## Introduction

Concerning the engineered or bacterial nucleases, the progress of genome editing machinery has provided the possibility of direct and specific recognition and modification of genomic sequences in practically all eukaryotic cells [1, 2]. Genome editing has resulted in the advancement of our knowledge respecting the finding of innovative therapeutic options for treating a wide spectrum of human disorders, ranging from infection to cancer. Current development in evolving programmable nucleases, including zinc finger nucleases (ZFNs), transcription activator-like effector nucleases (TALENs), as well as clustered regularly interspaced short palindromic repeat (CRISPR)-CRISPR-associated protein 9 (Cas9), has critically accelerated the development of gene editing from notion to clinical practice [3]. As CRISPR-Cas9 has been suggested as an encouraging tool for generating gene knockouts, its competence to offer capable gene editing in primary T cells presents a pronounced study tool to support a paradigm shift in T cell-based immunotherapies, more importantly, next-generation chimeric antigen receptor (CAR)-T cells [4].

CAR-T cell therapy includes the genetic modification of patients’ autologous T cells or allograft cells to efficiently express a CAR involving a fusion protein of a selected single-chain fragment variable (ScFV) from a specific monoclonal antibody and one or more T cell receptor intracellular signaling domains. This chimer receptor can selectively and efficiently recognize the related tumor-associated antigen (TAA) expressed by tumor cells [5]. Nonetheless, severe and life-threatening toxicities, such as cytokine releases syndrome (CRS), graft-versus-host disease (GVHD), on-target/off-tumor toxicity, neurotoxicity, and tumor lysis syndrome, commonly constrain its clinical utility [6]. Correspondingly, it seems that further progress in the next-generation CAR-T cells with more optimized construction, promoted efficacy, and moderated toxicities is of paramount importance. Meanwhile, the production of the universal “off-the-shelf” CAR-T cells from healthy donors can circumvent the restraints and possibly be a milestone in the future. For overcoming the GVHD occurrence and potent rejection upon CAR-T cell, CRISPR/Cas9-mediated ablation of the endogenous αβ T cell receptor (TCR) has resulted in a pronounced success in preclinical studies [7]. The endogenous αβ TCR on adoptively transferred donor lymphocytes can identify alloantigens in human leukocyte antigen (HLA) mismatched recipients, and thereby leads to the GVHD; on the other hand, detection of foreign HLA molecules on donor T cells can cause rejection [7]. Further, ablation of beta-2-microglobulin (β2M), a pivotal subunit of HLA-I proteins, can potently avert swift eradication of allogeneic T cells those express foreign HLA-I molecules.

Also, it has been suggested that dual blockade of programmed cell death protein 1 (PD1), lymphocyte activation gene 3 (LAG-3), or cytotoxic T lymphocyte-associated antigen-4 (CTLA-4) using genome editing technologies can sustain the improved T cell effector activities, facilitating an abrogation in tumor growth [8]. Moreover, knockout of diacylglycerol kinase (DGK), which metabolizes diacylglycerol to phosphatidic acid, using CRISPR/Cas9 supported CAR-T cell anti-tumor functions against U87MGvIII glioblastoma cell in vitro and xenografts [9].

Herein, we deliver a brief overview concerning the CAR-T cell-based therapy to treat human cancer, ranging from hematological malignancies to solid tumors. Also, we discuss recent findings respecting the application of genome editing platforms, in particular CRISP-Cas9, for potentiating the safety and efficacy of CAR-T cells in the context of tumor immunotherapy.

## CRISPR/Cas9 therapeutic application

Early in 1987, CRISPRs were firstly discovered in *E. coli* and after that in a large number of other bacteria species [10]. Various investigations in 2005 displayed their likenesses to phage DNA, and succeeding studies indicated that these sequences contribute to bacterial and archaea adaptive immune responses toward offending foreign DNA by stimulating the RNA-guided DNA cleavage [11]. Today, the CRISPR-Cas systems are largely categorized into two main classes according to the structural dissimilarity of the Cas genes and their construction shape [12]. Meanwhile, a class 1 CRISPR-Cas system involves multiple effector complexes, while a class 2 system includes only a single effector protein. To date, six CRISPR-Cas types and approximately 29 subtypes have been discovered [13, 14]. The most commonly employed subtype of CRISPR systems is the type II CRISPR/Cas9 system, enabling targeting specific DNA sequences by a single Cas protein from Streptococcus pyogenes (SpCas9) [15]. The CRISPR/Cas9 system consists of two main parts, including a single-stranded guide RNA (sgRNA) as a particular 17–23 base-pair (bp) sequence intended for specific identification of target DNA region in a sequence-specific style, and also a Cas9 endonuclease [15]. The sgRNA sequence is required to be trailed by a short DNA sequence upstream to facilitate efficient compatibilization with the Cas9 protein [16]. Correspondingly, the sgRNA causes a connection with a target sequence by Watson-Crick base pairing and Cas9 exactly cuts the DNA for establishing a DNA double-strand break (DSB) [16]. Upon the DSB, DNA-DSB repair tools start genome repair. The DSBs can be repaired by one of the two main appliances that largely rein almost all cell types and organisms, including homology-directed repair (HDR) and nonhomologous end-joining (NHEJ), leading to the targeted integration or gene disruptions, respectively [17].

The further description concerning detailed mechanism of the CRISPR-Cas9 function and parameters implicated in the determining its efficacy is beyond the scopes of this article, and thereby audiences are referred to the some excellent review in this context [18–20].

Compared to ZFN or TALEN tools, CRISPR-Cas9 is more suitable because of its flexibility and the capacity for multiple gene editing [21]. Indeed, endonuclease-based ZFN or TALEN technologies request the reengineering of a unique enzyme, which should be manufactured distinctly regarding each target sequence [21], but, as the nuclease protein Cas9 is the same in all cases, can be appropriately engineered to detect novel regions by varying the guide RNA sequences (sgRNA) [22]. Moreover, compared to CRISPR-Cas9, ZFNs and TALENs request much more labor and are more expensive. On the other hand, the unique competence of CRISPR/Cas9 to edit multiple loci concurrently signifies that this toll is easier, more efficient, and more scalable in comparison to the ZFNs and TALENs [23]. Thus, in the context of CAR-T cell-based targeted therapy, it is currently applicable to concurrently affect several genes and accomplish loss of function (LOF) of potentially any genetic or epigenetic target utilizing CRISPR-Cas9 [24].

## CAR construction

**Fig. 1:**
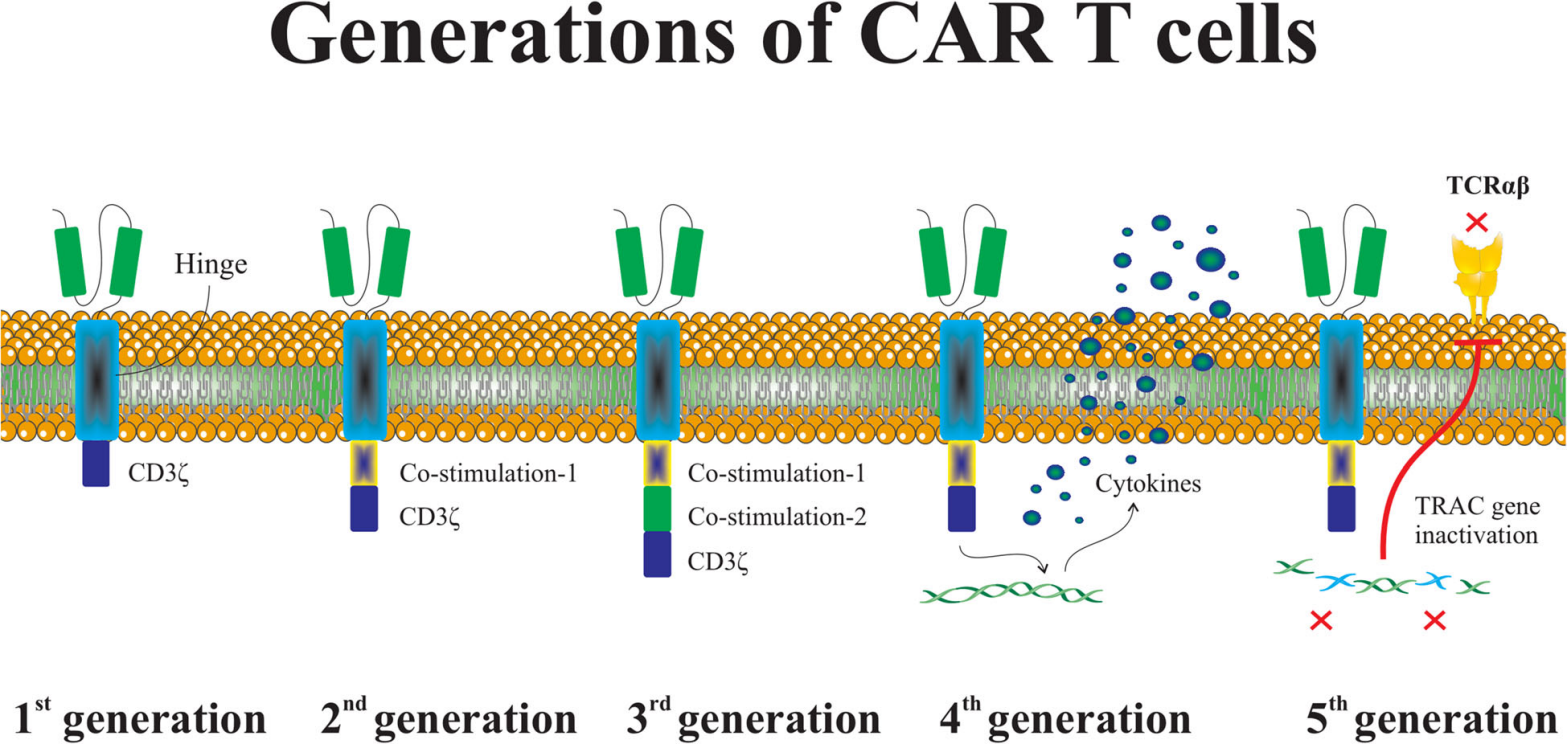
The basic structure of CAR-T cell generations. The first generation of CARs contains only a CD3ζ as a well-documented intracellular signal domain. The second and third generations of CARs involve one or two costimulatory molecules in addition to CD3ζ, respectively. As well, the fourth generation of CAR-T cells strongly motivates the downstream transcription factor to prompt cytokine generation following interrelation between CAR and target antigen. Prominently, the genome edition technologies, such as CRISPR-Cas9, have been widely used to construct TRAC (TCR)-deficient CAR-T cells, establishing fifth generation of CAR-T cells

Concisely, CAR is an engineered modified fusion protein structurally similar to the TCR and involves an extracellular antigen detecting domain linked to one or more intracellular signaling domains [5]. The CAR extracellular domain is structurally an antibody single-chain variable fragment (scFv) and identifies the target antigen virtually overexpressed on the tumor cells in the HLA-independent manner [25]. The CAR intracellular domains typically involve CD28, 4-1BB, or OX40 to support effector cell activation, and also include CD3ζ for the exertion of the cytotoxicity against transformed cells. The first generation of CARs involves only an intracellular signal domain CD3ζ, while the second generation of CARs includes a costimulatory molecule in addition to CD3ζ, and also the third generation of CARs contains another costimulatory domain. The recently advanced fourth generation of CAR-T cells could potently stimulate the downstream transcription factor to trigger cytokine release following the detection of the tumor-associated antigen (TAA) with CAR. Importantly, the fifth generation of CARs which has been constructed respecting the second generation utilizes gene editing to inhibit the expression of the TCR (TRAC) gene, facilitating the ablation of TCR alpha and beta chains (Fig. 1) [26]. As described, CRISPR system is widely used during the recent years to establish novel generation of CAR-T cells. T cells are engineered to generate transgenic cytokines, such as interleukin (IL-12) within the targeted tumor and therefore attract higher quantities of anti-tumor immune cells (e.g., natural killer (NK) cells and macrophages) to provide next-generation CAR-T cells for better toxicity management [27]. Moreover, CAR-T cells are equipped with chemokine receptors to circumvent their poor homing to tumor sites. These strategies like knocking in cytokines or chemokine receptors eventually augment CAR-T cell cytotoxic functions against tumor cells. As well, approaches like knocking out immune checkpoint molecules, and also ablation of TRAC or B2M can ameliorate CAR cell persistent in vivo and also enables CAR-T cell generation form allogeneic donors [28]. As well, knocking out the endogenous TGF-β receptor II (TGFBR2) in CAR-T cells using CRISPR/Cas9 method largely attenuates the elicited Treg conversion and thus hinders the exhaustion of CAR-T cells [29].

The CAR-bearing modified T cells can recognize CAR-targeted antigen and thus elicit T cell proliferation, cytokine manufacture, and critical and targeted cytotoxicity versus tumor cells [30]. Therefore, CAR-T cell treatment has supported appreciated attainment to treat hematological malignancies, including lymphoma, chronic lymphocytic leukemia (CLL), and acute lymphoblastic leukemia (ALL) [31, 32]. CARs deliver a wider array of functional impacts than transduced TCRs; however, CARs and TCRs have their advantages and disadvantages [33]. Although the flexibility and dynamic range of CARs are striking, existing CARs are restricted to identify cell surface antigens [33] while TCRs identify both cell surface and intracellular proteins. Nonetheless, antigen processing and presentation by HLA are not required for CARs, making them more applicable than TCRs to HLA-diverse patient populations [34].

The CAR’s engineering into T cells demands that T cells be cultivated to permit for transduction and succeeding expansion. Although the transduction can exploit diverse methods, steady gene transfer is essential to facilitate continued CAR expression in clonally expanding and persisting T cells.

## CAR-T cells generation from autologous and allogeneic T cells

The genetic alteration of autologous or allogeneic peripheral blood T lymphocytes to create tumor-targeted T cells has become an inspiring therapeutic option. The great and pronounced competencies of TCR and CAR therapies are best exemplified through the stimulating clinical results achieved with NY-ESO-1 TCR [35] and CD19 CAR-T cells [36, 37]. CAR-T cell construction processes combine T cell activation and transduction stages for providing genetically targeted T cell products. Indeed, engineered T cells to express particular CARs can be generated from Ficoll-purified PBMCs followed by their activation with anti-CD3 monoclonal antibody (mAb) in the existence of irradiated allogeneic feeder cells, and finally efficient transduction with a vector encoding the CAR [38]. The encouraging clinical outcomes of CAR-T cell therapy may be more enlarged by establishing the potent and histocompatible T cells. Autologous methods have a confirmed track record, but personalized products can be challenging in some cases, for instance in patients with chemotherapy or HIV-mediated immune deficiency [39]. Accordingly, though T cells can be simply achieved from donors, their application is potently hindered by the high alloreactive capability. Indeed, TCRs have the natural competence to respond toward non-autologous tissues, identifying both allogeneic HLA molecules and other minor antigens [40]. This tendency inspires the incidence of graft rejection in transplant recipients and also the occurrence of GVHD in recipients of donor-isolated T cells [41]. Given these problems, inhibition of the alloreactive potential of allogeneic T cells to obtain an acceptable risk-benefit ratio is of paramount importance. To date, two main tactics have been designed to defeat the risk of graft-versus-host reaction (GVHR) concerning the selection of virus-specific TCRs devoid of GVHR or the ablation of TCR expression [39]. As described, three main technologies, containing ZFNs, TALEN, and CRISPR/Cas9, facilitate gene disruption in the human cell. Remarkably, the ablation of endogenous TCR expression largely obtained through utilizing genome-editing technologies abrogate the continuous districts of TRAC genes, and thereby offer the chance for manufacturing universal CAR-T cells [7, 42].

To CAR-T cells hold potential as a safe and rapidly evolving therapeutic strategy for treating human malignancies, the development of methods to pharmacologically control them in vivo is required. Owing to this fact, some strategies, in particular, suicide mechanisms are developing [43, 44]. For example, Amatya and her colleagues designed a construction including CD28-containing anti-signaling lymphocytic activation molecule F7 (SLAMF7) CAR and a suicide gene [45]. SLAMF7 is a capable target for CAR-T cell treatment of multiple myeloma (MM) because of their robust expression on the surface of MM but not normal nonhematopoietic cells. The suicide gene encoded a dimerization domain bonded to a caspase-9 domain [45]. They showed that T cells expressing the SLAMF7-specific CAR accompanied with suicide-gene construct specifically identified and eradicated SLAMF7-positive cells in vitro and tumor cell-bearing mice. Interestingly, engineered T cells were eradicated on demand through injection of the dimerizing agent AP1903 [45]. However, as suicide strategies mainly result in the complete elimination of the CAT-T cells, they will possibly lead to the premature end of the intervention. Consequently, carrying out non-lethal control of CAR-T cells is required to expand the CAR-T cell both efficacy and safety [46]. In this regard, small molecule-based plans as described by Lim et al. can offer a possibility to turn the CAR-T cells “on” or “off” [47]. Further, synthetic splitting receptor [46], combinatorial target-antigen recognition [48], synthetic Notch receptors [49], and bispecific T cell engager [50] along with inhibitory chimeric antigen receptor (iCAR) [51] are other suggested strategies for improving the safety of engineered T cell.

## CAR-T cell in clinical trails

**Fig. 2:**
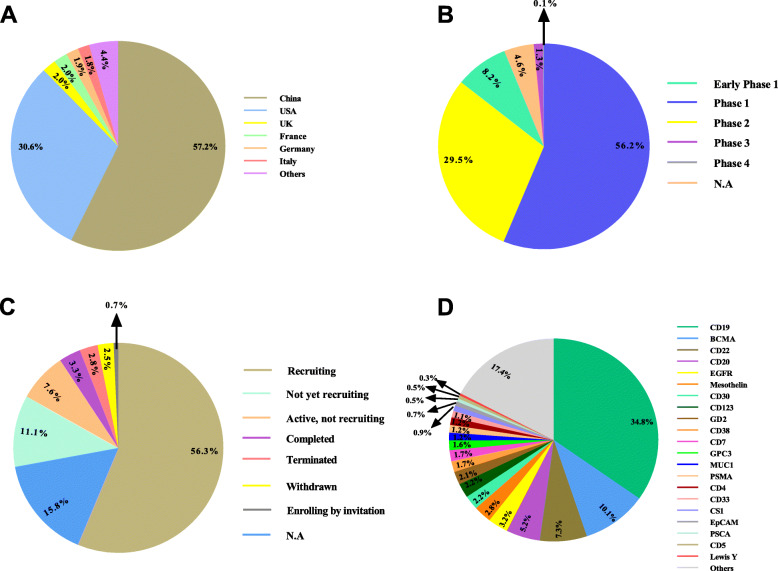
Clinical trials concerning the CRISPR-Cas9 application in the context of CAR-T cell-based tumor immunotherapy registered in ClinicalTrials.gov (June 2021). The schematic presents conducted or ongoing clinical trials based on the CRISPR-Cas9-mediated genome edition in CAR-T cell depending on the study location (**A**), study phase (**B**), study status (**C**), and target antigen (**D**) registered in ClinicalTrials.gov (June 2021)

Valuing the hopeful results achieved from a myriad of preclinical studies, numerous clinical trials have been conducted or are ongoing to address the safety, feasibility, and efficacy of CAR-T cells in patients suffering from hematological malignancies or solid tumors (Fig. 2) (Table 1).

**Table 1:** Phase 2 and 3 clinical trials based on the CAR-T cell therapy in the context of the tumor immunotherapy registered in ClinicalTrials.gov (June 2021)

Condition	Target antigen	Phase	Participant Number	Location	Status	NCT number
Lymphoma	CD19	2	78	France	Recruiting	NCT04703686
MM	BCMA	2	60	China	Active, not recruiting	NCT03758417
AML	CD123	2/3	20	China	Recruiting	NCT03631576
B-ALL	CD19	2/3	10	Malaysia	Recruiting	NCT03937544
B cell leukemia/lymphoma	CD19	2	25	Sweden	Active, not recruiting	NCT03068416
B-ALL	CD19,CD22	1/2	20	China	Recruiting	NCT04723901
B cell lymphoma	CD19,CD20	1/2	20	China	Recruiting	NCT04723914
Leukemia or lymphoma	CD19	1/2	16	USA	Active, not recruiting	NCT03684889
NHL	CD19,CD20	1/2	30	China	Recruiting	NCT04697940
Gastric and pancreatic cancers	Claudin	1/2	102	China	Recruiting	NCT04581473
NSCLC	MUC1	1/2	60	China	Recruiting	NCT03525782
AML	CLL1,CD33,CD123	1/2	10	China	Recruiting	NCT04010877
MM	SLAMF7	1/2	38	Germany	Recruiting	NCT04499339
B cell lymphoma	CD19	1/2	11	China	Recruiting	NCT04429438
B-ALL	CD19	1/2	185	Germany	Recruiting	NCT04404660
Ovarian cancer	MESO	1/2	20	China	Recruiting	NCT03916679
AML and MM	CD44v6	1/2	58	Italy	Recruiting	NCT04097301
Sarcoma	CD133, GD2, MUC1, CD11	1/2	20	China	Recruiting	NCT03356782
B-ALL	CD19	1/2	15	Sweden	Completed	NCT02132624
MM	CD38 , BCMA	1/2	80	China	Recruiting	NCT03767751
B-All	CD19 , CD22	1/2	23	UK	Completed	NCT03289455
MM	BCMA	1/2	220	USA	Recruiting	NCT03288493
MCL	CD19	2	59	China	Recruiting	NCT04718883
AML	CD33, CD123,CLL-1	1/2	10	China	Recruiting	NCT04010877
B-ALL and B-NHL	CD19	2	90	USA	Recruiting	NCT04148430
MCL	CD19	2	36	USA	Recruiting	NCT04484012
HL	CD30	2	94	USA	Recruiting	NCT04268706
ALL and NHL	CD19	1/2	32	Italy	Recruiting	NCT03373071
Neuroblastoma	GD2	1/2	42	Italy	Recruiting	NCT03373097
HL	CD30	1/2	30	Spain	Recruiting	NCT04653649
Solid tumors	PSMA	1/2	100	China	Recruiting	NCT04429451
B cell lymphoma	CD19	1/2	43	USA	Active, not recruiting	NCT00924326
ALL and NHL	CD19	1/2	24	Turkey	Recruiting	NCT04206943
B cell lymphoma	CD19	1/2	20	USA	Recruiting	NCT04257578
B cell lymphoma	CD19	1/2	1	USA	Completed	NCT01475058
Solid tumors	Mesothelin	1/2	15	USA	Terminated	NCT01583686
Melanoma and renal cancers	VEGFR2	1/2	24	USA	Terminated	NCT01218867
NHL	CD19,CD20	1/2	80	China	Recruiting	NCT04553393
Pancreatic and prostate cancer	PSCA	1/2	151	USA	Recruiting	NCT02744287
Leukemia	CD19	1/2	177	China	Completed	NCT03173417
ALL	CD22	2	100	China	Recruiting	NCT04340167
B cell leukemia or lymphoma	CD19, CD20	1/2	100	China	Completed	NCT03097770
Esophageal cancer	PD1**,** MUC1	1/2	20	China	Recruiting	NCT03706326
NHL MCL	CD19, CD20	1/2	32	USA	Recruiting	NCT04186520
B cell leukemia/ lymphoma	CD19, CD22	1/2	40	China	Recruiting	NCT04648475
B cell leukemia/ lymphoma	CD22	1/2	42	USA	Recruiting	NCT04571138
Acute leukemia	CD19	1/2	167	USA	Active, not recruiting	NCT02028455
B-ALL	CD19	1/2	18	Russian	Active, not recruiting	NCT03467256
B-ALL and B-NHL	CD19	1/2	50	USA	Recruiting	NCT04544592
ALL and NHL	CD19	1/2	60	Canada	Recruiting	NCT03765177
MM	BCMA	1/2	30	USA	Recruiting	NCT03448978
ALL	CD19	1/2	35	USA	Recruiting	NCT03573700
Pancreatic and prostate cancer	PSCA	1/2	151	USA	Recruiting	NCT02744287
B cell leukemia/ lymphoma	CD19,CD22	1/2	30	USA	Not yet recruiting	NCT04029038
B cell leukemia/ lymphoma	CD19,CD22	1/2	40	China	Recruiting	NCT04649983
Acute leukemia	CD19, BCMA	1/2	20	China	Recruiting	NCT04846439
Brain tumors	EGFRvIII	1/2	18	USA	Completed	NCT01454596
ALL and NHL	CD19	1/2	24	Turkey	Recruiting	NCT04206943
B cell malignancies	CD19, CD20, CD22 CD30, CD38, CD70, CD123	1/2	100	China	Recruiting	NCT03125577
B-ALL	CD19	2	82	USA	Terminated	NCT02535364
DLBCL	CD19	2	115	USA	Active, not recruiting	NCT02445248
Adult large B cell lymphoma	CD19	1/2	91	South Korea	Recruiting	NCT04836507
DLBCL	CD19	2	25	USA	Terminated	NCT03954106
Solid tumors	NY-ESO-1	1/2	50	China	Recruiting	NCT03941626
B- ALL and B-NHL	CD19	1/2	300	Israel	Recruiting	NCT02772198
ALL, DLBCL and PML	CD19	1/2	32	Italy	Recruiting	NCT04787263
B cell lymphoma	CD19	4	10	China	Not yet recruiting	NCT02992834
NHL and ALL	CD19	1/2	63	Canada	Recruiting	NCT03938987
AML	CD33	1/2	34	USA	Recruiting	NCT03971799
ALL, NHL, CLL, DLBCL, FL MCL	CD19	1/2	48	Germany	Recruiting	NCT03676504
Glioblastoma	B7-H3 (CD276)	1/2	40	China	Recruiting	NCT04077866
AML and CLL	CD19	1/2	28	China	Completed	NCT03076437
MM	BCMA	2	120	USA	Recruiting	NCT04133636
DLBCL, FL and MCL	CD19	1/2	12	USA	Active, not recruiting	NCT02650999
B cell malignancy	CD19,CD20	1/2	100	China	Completed	NCT03097770
T-ALL, T-NHL and AML	CD7	1/2	108	China	Recruiting	NCT04599556
Esophageal cancer	MUC1,PD-1	1/2	20	China	Recruiting	NCT03706326
NHL and MCL	CD19,CD20	1/2	32	USA	Recruiting	NCT04186520
Leukemia/lymphoma	CD22	1/2	42	USA	Recruiting	NCT04571138
Cervical cancer	GD2, PSMA, MUC1, Mesothelin	1/2	20	China	Recruiting	NCT03356795
Acute leukemia	CD19	1/2	167	USA	Active, not recruiting	NCT02028455
B-ALL	CD19	1/2	18	Russian	Active, not recruiting	NCT03467256
B-ALL and B-NHL	CD19	1/2	50	USA	Recruiting	NCT04544592
ALL and NHL	CD19	1/2	60	Canada	Recruiting	NCT03765177
HL and NHL	CD30	1/2	40	USA	Recruiting	NCT02690545
MM	BCMA	1/2	30	USA	Recruiting	NCT03448978
T cell lymphoma	CD30	2	20	USA	Recruiting	NCT04083495
Solid tumors	Mesothelin	1/2	179	USA	Recruiting	NCT02414269
B- NHL	CD19	2	61	USA	Active, not recruiting	NCT03483103

Note: *ALL* acute lymphoblastic leukemia, *NHL* non-Hodgkin’s lymphoma, *AML* acute myeloid leukemia, *HL* Hodgkin lymphoma, *BCMA* B cell maturation antigen, *MM* multiple myeloma, *MCL* mantle cell lymphoma, *DLBCL* diffuse large B cell lymphoma, *CLL* chronic lymphocytic leukemia, *FL* follicular lymphoma, *PSMA* prostate-specific membrane antigen, *PSCA* prostate stem cell antigen, *SLAMF7* signaling lymphocytic activation molecule F7

### Hematological malignancies

Anti-CD19 CAR-T cell therapy has presented notable activity in patients with refractory or relapsed acute lymphocytic leukemia (ALL). Several anti-CD19 CAR-T cell constructs have been investigated and responses differ extensively among various studies [52]. In 2017, the Food and Drug Administration (FDA) granted regular approval to axicabtagene ciloleucel or Yescarta as a therapeutic option for large B cell lymphoma (BCL). Yescarta is a CD19-specific CAR-T cell mainly exploited for the treatment of adult patients with relapsed or refractory large BCL following two or more lines of systemic treatment. However, a trial in 101 patients with BCL who received a single injection of axicabtagene ciloleucel followed by lymphodepleting chemotherapy using cyclophosphamide and fludarabine indicated that intervention led to severe unwanted events in 52% of participants. Also, recurrence of the CRS and neurologic toxicities in 94% and 87% of participants, respectively, signified the importance of the operation of a risk assessment and mitigation strategy [53]. Nonetheless, infusion of the axicabtagene ciloleucel to 111 participants with diffuse large B cell lymphoma (DLBCL) at the dosage of 2 × 10^6^ CD19-CAR-T cells/kg displayed significant efficacy. While the complete response rate was 54%, a significant number of patients experienced neutropenia, anemia accompanied by thrombocytopenia. Also, 13% and 28% of the patients experienced robust CRS and neurological effects, respectively [54]. Furthermore, brexucabtagene autoleucel (KTE-X19), another CD3ζ/CD28-based CD19-specific CAR-T cell, is specified for mantle cell lymphoma (MCL) therapy. A phase 2 trial in 74 participants with relapsed or refractory MCL revealed that brexucabtagene autoleucel could elicit durable remissions in a majority of patients who received 2 × 10^6^ CD19-CAR-T cells/kg. However, similar to the previous reports, the intervention exerted severe and life-threatening toxic influences [55]. As well, KTE-C19 as an autologous CD3ζ/CD28-based CD19-specific CAR-T cell product at a target dose of 2 × 10^6^ CAR-T cells/kg showed an acceptable safety profile along with an overall response rate of about 71%, and a complete response rate of about 57% in a participant with refractory DLBCL [56]. On the other hand, anti-B cell maturation antigen (BCMA) CAR-T cell therapy has been revealed to have desired activities in patients with relapsed or refractory multiple myeloma (MM) [57]. As well, a small subgroup of MM cells typically express CD19, and thereby CD19-CAR-T cell therapy has displayed a positive anti-tumor effect in some of these patients [57]. Evaluation of the safety and efficacy of combined treatment with anti-CD19 and anti-BCMA CAR-T cells in participants with relapsed or refractory MM have indicated that administration of humanized CD19-CAR-T cells accompanied by murine BCMA CAR-T cells at the similar dosage of 1 × 106 cells/kg following lymphocyte depletion may result in significant preliminary activity. But, the intervention led to the higher unwanted events, containing neutropenia, anemia, and thrombocytopenia in 86%, 62%, and 62% of enrolled participants, respectively, concomitant with one intervention-related death possibly due to the thrombocytopenia [57]. Besides, tisagenlecleucel, an autologous T cell with a lentiviral vector encoding a CD19-specific CAR, presented a significant efficacy along with a manageable safety profile in a subgroup of Japanese patients with relapsed/refractory (r/r) B-ALL [58] and DLBCL [59], making them a rational treatment strategy in patients with B-ALL and DLBCL.

In addition to the cited trails, a myriad of trials based on the targeting BCMA in MM ([60–65], CD19 in ALL [32, 66–74] and non-Hodgkin’s lymphoma (NHL) [69, 75–79], CD20 in BCL [70, 80–82], and CD22 in ALL [83–86] have shown the significant efficacy in the clinic.

### Solid tumors

CAR-T cell therapy is more restricted in solid tumors than in hematological malignancies as CAR-T cells are circulated to the bloodstream and lymphatic system, and thereby have more interaction with blood tumor cells. Nevertheless, in solid tumors, these redirected effector cells may not be able to penetrate tumor tissue by the vascular endothelium [87]. Overall, studies have recognized various roadblocks for administered CAR-T cells, comprising a restricted spectrum of targetable antigens and heterogeneous antigen expression, restricted T cell survival before reaching tumor region, incapability of T cells to proficiently recruit to tumor region and penetrate physical barriers, and finally an immunosuppressive TME [88]. Nonetheless, various tumor-associated antigens (TAA) have been targeted by redirected effector immune cells to elicit an anti-tumor response in vitro and in vivo. For instance, anti-prostate-specific membrane antigen (PSMA) CAR-T cells could selectively target PSMA-positive cells in vitro and eradicate tumor cells in vivo [89]. A trial in 6 patients with prostate cancer revealed that infusion of the PSMA-specific autologous CAR-T cell led to no anti-PSMA toxicities and reactivities. Moreover, the use of PSMA-specific CAR-T cell plus IL-2 resulted in more prominent anti-tumor responses than monotherapy and thereby suggested that pharmacodynamics of “drug-drug” interactions could improve the efficacy of their co-application [90]. Further, it has been found that the potent activity of anti-PSMA CAR-T cells could be improved through the co-expression of a dominant-negative TGF-βRII (dnTGF-βRII). Meanwhile, expression of the dominant-negative TGF-βRII in CAR-T cells could support improved lymphocyte proliferation, augmented cytokine secretion, resistance to exhaustion, prolonged in vivo persistence, and also the stimulation of tumor elimination in vivo. As well, this strategy could be effective for the treatment of patients suffering from relapsed and refractory metastatic prostate cancer [91]. Interestingly, combine treatment with GD2 specific CAR-T cell with CD3ζ, CD28, and OX40 signaling domains and pembrolizumab (anti-PD-1 mAb) may augment the anti-tumor activity of the effector T cells by improving their persistence and expansion in patients with GD2-positive tumors, such as melanoma [92]. On the other hand, constructing and injecting anti-EGFRvIII CAR-T cells is feasible and safe, without indication of off-tumor toxicity or CRS [93, 94]. However, systemic injection of a single dose of EGFRvIII-specific CAR-T cells into 10 patients with glioblastoma mediated antigen loss and stimulated adaptive resistance in patients with recurrent glioblastoma [93]. These findings have shown that while systemic infusion could support on-target effect in the brain, defeating the adaptive variations in the local TME concurrently addressing the antigen heterogeneity are required to improve EGFRvIII-directed approaches in glioblastoma [93]. Moreover, a phase I/II clinical study in 19 patients with recurrent/refractory human epidermal growth factor receptor 2 (HER2)-positive sarcoma showed that injections were well tolerated in the lack of no dose-limiting toxicity [95]. This study was the first trial of the safety and efficacy of HER2-CAR-T cells in patients with tumors showing that the administrated cells persisted for 6 weeks without obvious toxicities [95]. Similarly, the safety and feasibility of HER2-CAR-T cell therapy were shown in patients with advanced biliary tract cancers (BTCs) and pancreatic cancers [96]. Besides, transplantation of the carboxy-anhydrase-IX (CAIX)-specific CAR-T cell into 12 patients with CAIX-expressing metastatic renal cell carcinoma (RCC) delivered in-patient proof that intervention could lead to positive anti-tumor responses [97].

In addition to the listed reports, CAR-T cell therapy based on the targeting tumor-associated glycoprotein (TAG)-72 in colorectal cancer [98], carcinoembryonic antigen (CEA) in lung cancer [99] and liver cancer [100], mesothelin [101], and EGFR [102] in pancreatic cancer, fibroblast activation protein (FAP) in mesothelioma [103], IL13Rα2 in glioblastoma [104], and mucin-1 (MUC1) in seminal vesicle cancer [105] have been conducted or are ongoing to address the safety and efficacy of redirected effector T cells in patients with tumors.

## CRISPR/Cas9 potential to overcome potent challenges of CAR-T cell-based therapies

Currently, CRISPR/Cas9-mediated genome editing offers the potential of more effective immunotherapy, by manufacturing a universal “off-the-shelf” cellular product or modifying immune cells to defeat resistance in hematological or solid tumors (Table 2). Despite the existence of several challenges concerning the safety, efficiency, and scalability of this strategy, the CRISPR/Cas9 approach will undeniably reign in the context of CAR-T cell-based therapies for tumors [119].

**Table 2:** Preclinical studies based on the use of CRISPR-Cas9 technology to provide more effective and universal CAR-T cell

Condition	CAR	Target locus (knocked out)	Study model	Ref
ALL	CD19	Pax5 Ebf1	C57Bl/6 mice	[106]
ALL	CD19	LDLR	NSG mice	[107]
Ewing sarcoma	Ganglioside G(D2)	EZH2	VH-64, RM-82, and WE-68 cell lines NSG mice	[108]
Glioma	EGFRvIII	DGK	U87 MG line NSG mice	[9]
Liver cancer Ovarian cancer	Mesothelin	TGF-βRII	HepG2 , and OVCAR3 cell line NPG mice	[29]
ALL Prostate cancer	PSCA CD19	TRAC B2M PD1	NSG mice	[109]
Glioma	CD133	PD1	U251 cell line NPG mice	[110]
Glioma	EGFRvIII	TRAC B2M PD1	U87 and U251 cell line NSG mice	[111]
Glioma	EGFRvIII	PD1	U251 cell line	[24]
BCL	CD19	LAG-3	NSG mice	[112]
BCL	CD22	TRAC PD-1	NALM6 cell line	[113]
BCL	BCMA CD19	TRAC	Cell line	[7]
ALL	CD7	TRAC	MOLT-3, MOLT-4, HSB-2, and CCRF-CEM cell line NSG mice	[114]
ALL	CD19	GM-CSF	Cell line NSG mice	[115]
ALL	CD19	GM-CSF	NALM6 and MOLM13 cell line NSG mice	[116]
BCL	CD19	TRAC	NSG mice	[117]
ALL	CD19	TRAC	NSG mice	[118]

Note: *ALL* acute lymphoblastic leukemia, *BCL* B cell lymphoma, *EGFR* vIII epidermal growth factor receptor variant III, *PSCA* prostate stem cell antigen, *BCMA* B cell maturation antigen, *PAX5* paired box 5, *EBF1* EBF transcription factor 1, *LDLR* low-density lipoprotein receptor, *EZH2* enhancer of zeste homolog 2, *DGK* diacylglycerol kinase, *TGF-βRII* transforming growth factor beta receptor II, *TRAC* T cell receptor alpha constant, *B2M* beta-2-microglobulin, *PDCD1 or PD1* programmed cell death protein 1, *LAG-3* lymphocyte activation gene 3, *GM-CSF* granulocyte-macrophage colony-stimulating factor

### Disruption of inhibitory molecules and signaling axis

It has been suggested that merging lentiviral delivery of CAR and electro-transfer of Cas9 mRNA and gRNAs targeting endogenous TCR, β-2 B2M, and PD-1 simultaneously cause preparing the universal “off-the-shelf” CAR-T cells. Meanwhile, TCR and HLA class I double-deficient T cells potentially show diminished alloreactivity and commonly cause no GVHD [109, 120]. Moreover, concurrent triple genome editing could support ameliorated in vivo anticancer functions of the gene-disrupted redirected effector T cells [109, 120]. Similarly, triple gene-disrupted CAR-T cells displayed raised activity in glioma mice models leading to the extended overall survival rate in mice bearing intracranial tumors following intracerebral, but not systemic administration [24]. Moreover, marked PD-1 gene disruption lonely can be an attractive plan to enhance the efficacy of CAR-T cell therapy in an immunosuppressive TME [110]. Hu et al. found that PD-1 gene disruption by CRISPR/Cas9 and using piggyBac transposon system for expressing CD133-specific CAR in one reaction resulted in the comparable rates of cytokine releases, while led to the promoted growth and cytotoxicity in vitro. Also, engineered CAR-T cells displayed robust resistance to inhibitory molecules in the glioma murine model compared to conventional CD133-CAR-T cells [110]. Likewise, PD-1-disrupted EGFRvIII-specific CAR-T cells exerted evident suppressive impacts in vitro on EGFRvIII positive glioblastoma cells (U-251MG and EGFRvIII-expressing DKMG) without any significant influence on the T cell phenotype and the expression of other checkpoint receptors [111]. Thereby, Nakazawa et al. suggested that the sgRNA/Cas9-mediated anti-tumor activities of EGFRvIII-specific CAR-T cells are intensely dependent on PD-1 disruption [111]. Besides, PD-1-deficient CD19-specific CAR-T cells showed elevated anti-tumor activity against and improved clearance of CD19+ PD-L1+ K562 myelogenous leukemia cells in NOD-SCID-IL-2Rγ−/− (NSG) mice compared to the conventional CD19-specific CAR-T cell [121]. Albeit, it was found that ectopic PD-L1 expression could not significantly modify intrinsic tumor proliferation in K562 cell-bearing mice since there was no alteration in growth kinetics of CD19+ and CD19+ PD-L1+ cells in the experimental model [121]. Too, PD-1 deficient mesothelin-specific CAR-T cell diminished PD-1+ population in triple-negative breast cancer (TNBC) [122]. Although observed attenuation had no significant impact on CAR-T cell proliferation, it stimulated CAR-T cell cytokine generation and cytotoxicity against PD-L1-expressing TNBC cells in vitro. More efficiently, PD-1 deficient mesothelin-specific CAR-T cells demonstrated a more prominent effect on tumor control and relapse prevention in the preclinical model than conventional CAR-T cells [122]. Besides, lymphocyte activation gene-3 (LAG-3) knockout CD19-specific CAR-T cells by CRISPR-Cas9 elicited strong antigen-specific anti-tumor effects in vitro and lymphoma Raji cell-bearing NOD-Prkdcscid Il2rgnull (NPG) mice. Nonetheless, LAG-3 knockout CAR-T cells showed no superiority in terms of the anti-tumor response and the reduction in tumor burden compared to the conventional CAR-T cells [112].

### Reducing CRS and GVHD occurrence

As described, TCR and HLA class I double-deficient CAR-T cells robustly display attenuated alloreactivity and universally result in no GVHD occurrence. As well, these cells’ anti-tumor activity can be potently intensified by simultaneous ablation of PD-1 and CTLA-4 [123]. It has been documented that fratricide-resistant “off-the-shelf” CAR-T, known as UCART7, as a novel anti-CD7 CAR-T cell with a deficiency in TCR could exert robust cytotoxicity against CD7 expressing malignant cells in vitro and in vivo without GVHD development. Both UCART7 and anti-CD7 CAR-T cells could detect and eliminate CD7+ leukemic cell lines, MOLT3, CCRF-CEM, and HSB-2 in vitro with similar efficiencies, representative of no impairment in activity upon double deletion of CD7 and TCR [114]. Thereby, UCART7 as an allo-tolerant “off-the-shelf” CAR-T cell product signifies an efficient and applicable option for treating the relapsed and refractory T-ALL and non-Hodgkin’s T cell lymphoma [114].

Given the importance of the granulocyte-macrophage colony-stimulating factor (GM-CSF) in the simulation of CRS, some studies have focused on the attenuation of its effect on the CRS induction upon CAR-T cell therapy. GM-CSF is a colony-stimulating factor that adjusts the proliferation and differentiation of hematopoietic cells. This cytokine is abundantly generated by CAR-T cells following activation and exists in the TME at high levels [124]. In 2019, Sterner et al. investigated the use of CRISPR/Cas9 gene editing in CD19-specific CAR-T cells by transduction with a lentiviral construct including a guide RNA to GM-CSF and Cas9 [115]. They found that GM-CSF deficient anti-CD19 CAR-T cells efficiently released less GM-CSF, whereas maintained pivotal T cell function. Importantly, these redirected effector T cells exhibited a more prominent anti-tumor effect than wild-type CAR-T cells in vivo [115]. In another study, they found that GM-CSF neutralization with lenzilumab did not elicit any negative effect on anti-CD19 CAR-T cell activity in vitro and in vivo. Furthermore, anti-CD19 CAR-T cell proliferation was improved and durable control of ALL was ameliorated in patient-derived xenografts following GM-CSF neutralization with lenzilumab [116]. Finally, they found that GM-CSF deficient CAR-T cells upheld normal activity and had a superior anti-tumor function in vivo leading to an improved overall survival rate in comparison to the conventional anti-CD19 CAR-T cell [116].

### Manufacturing allogeneic universal CAR-T cells

It is mainly difficult in newborn and elder patients to achieve sufficient and good quality T cells for manufacturing the patient-specific CAR-T cells. For providing more accessible CAR-T cells, it is greatly wanted to progress an allogeneic adoptive transfer plan, in which universal CAR-T cells are produced from healthy donor’s T cells to treat numerous patients [123, 125].

As cited, allogeneic universal CAR-T cells can potently be established by impairing TCR and B2M gene expression in CAR-T cells by genome editing strategies. Correspondingly, CAR+TCR^_^T cells seem to be a rational approach to introduce as the new generation CAR-T cell, providing an “off-the-shelf” therapy for the tentative treatment of B-lineage malignancies [114]. Genetically edition of anti-CD19 CAR-T cells to disrupt expression of the endogenous TCR for inhibition of GVHD progress could display the anticipated property of conventional CD19-specific CAR-T cells without responding to TCR stimulation [126]. Likewise, another report has implied that directing CD19-specific CAR to the TCR locus may sustain the uniform CAR expression in T cells and simultaneously improve T cell potency [117]. Remarkably, Eyquem et al. found that TCR-deficient CD19-specific CAR-T cells could trigger better anti-tumor response compared to conventional CAR-T cells in a mice model of ALL [117]. In addition, directing the CAR to the TCR locus prevents tonic CAR signaling and enables effective internalization and re-expression of the CAR upon the single or repeated exposure to antigen, which in turn leads to the delayed effector T cell differentiation and exhaustion. Indeed, targeting CARs to a TCR locus offers a safer therapeutic T cell by reducing the risk of insertional oncogenesis and TCR-stimulated autoimmunity and alloreactivity in addition to providing a more potent T cell, as documented by minimizing the constitutive signaling and abrogation of T cell depletion [117].

### Resistance to the suppressive effects of TGF-β

Despite CAR-T cells’ remarkable activity against cancer, this therapeutic option still faces various challenges, in particular, immunosuppressive tumor microenvironment (TME) for eradicating solid tumors [29]. Although TGF-β exerts tumor-suppressive influences through inhibiting cell cycle development and inducing apoptosis in the early stages of tumors, TGF-β elicits tumor-promoting influences leading to the boosted tumor invasiveness as well as metastasis in late stages [127]. Besides, the TGF-β signaling axis creates interactions with other signaling axes in a synergistic or antagonistic mode and controls biological procedures. Taken together, given the critical role of TGF-β in tumor progress, this pathway is a rational target for tumor therapy. Various therapeutic strategies, comprising TGF-β antibodies, antisense oligonucleotides, and small molecules inhibitors of TGF-β receptor-1 (TGF-βR1), have exposed huge competence to negatively regulate TGF-β signaling [127].

It has been robustly evidenced that suppression of TGF-βR signaling improves the anti-tumor activities of receptor tyrosine kinase-like orphan receptor 1 (ROR1)-specific CAR-T cells toward TNBC. Meanwhile, blockade of the TGF-βR axis using the specific inhibitors could largely protect CD8+ and CD4+ ROR1-CAR-T cells from the suppressive impacts of TGF-β, facilitating their tumor-suppressive activity in the 3D tumor model [29]. Similarly, dominant-negative TGF-βR promotes PSMA-specific CAR-T cell proliferation and strongly increases prostate cancer elimination. These CAR-T cells demonstrate improved cytokine generation, resistance to exhaustion, and also prolonged persistence in vivo [91]. Moreover, the knocking out of the endogenous TGF-β receptor II (TGFBR2) in anti-mesothelin CAR-T cells using the CRISPR/Cas9 technique may decrease the activated Treg conversion and avoid CAR-T cells depletion [29]. Importantly, TGFBR2-edited CAR-T cells exhibited a more obvious capability to eliminate mesothelin-expressing CRL5826 and OVCAR-3 cells in tumor cell-bearing mice when injected locally or systemically [29]. As well, TGF-βRII-edited CAR-T cells are mainly resistant to TGF-β inhibition, and also elicit augmented cell killing compared to the conventional CAR-T cells in the existence of TGF-β against B cell maturation antigen (BCMA)-positive tumor cells [128]. Furthermore, CRISPR/Cas9-mediated knockout of the DGK, as a possible regulator of TGF-β, boosts the anti-tumor activity of the CAR-T versus U87MGvIII glioblastoma cell in vitro and murine models mainly by the triggering resistance to TGF-β and also PGE2 [9].

In addition to the CRISPR-Cas9 technology, other well-known genome-editing techniques have shown the pronounced capability to support the broader application of CAR-T cells (Table 3).

**Table 3:** Preclinical studies based on the use of TALEN and ZFN technologies to provide more effective and universal CAR-T cell

Condition	CAR	Target locus (knocked out)	*Study model*	*Ref*
*TALEN*				
MM	BCMA	CD20	MM.1S cell line NSG mice	[129]
B-ALL	CD19	TRAC CD52	NSG mice	[130, 131]
BL	CD22	TRAC CD25 PD-1	RAJI cell line NSG mice	[132]
T-ALL	CD3	TRAC	Jurkat cell line NSG mice	[133]
B-ALL	CD20	TRAC PD-1	Cell line	[134]
BL	CD22	GM-CSF	RAJI and Daudi cell line	[135]
B-ALL	CD19	TRAC CD52	NALM6 cell line NSG mice	[136]
*ZFN*				
B-ALL CLL MCL	CD19	TRAC	Primary tumor cells	[126]
B-ALL	CD19	TRAC	Cell line	[7]

Note: *ALL* acute lymphoblastic leukemia, *BCMA* B cell maturation antigen, *TRAC* T cell receptor alpha constant, *PDCD1 or PD1* programmed cell death protein 1, *GM-CSF* granulocyte-macrophage colony-stimulating factor, *MM* multiple myeloma, *MCL* mantle cell lymphoma, *BL* Burkitt’s lymphoma

## The off-target effects of CRISPR-Cas9 technology

Several classes of CRISPR-Cas systems have yet been advanced, while their comprehensive use can be hindered via off-target effects. Efforts are being accomplished to attenuate the off-target effects of CRISPR-Cas9 through establishing the multiple CRISPR/Cas systems with high fidelity and accuracy [137]. Thereby, a myriad of techniques have been utilized to identify off-target mutations, and restore the on-target effects and conversely reduce potent off-target effects. As the genomic frameworks of the targeted DNA concurrently the secondary structure of sgRNAs and their GC content are mainly contribute to determining cleavage efficiency, designing of the appropriate sgRNAs with high on-target activities using specific tools is severally suggested [137]. Recently, the amelioration of the specificity [138] of genome editing tools and the identification [139] of off-target effects are swiftly developing research areas. Such research incorporates designer nuclease development [140], discovery computational prediction programs and also databases [141] and also finding high-throughput sequencing [139] to diminish mutational occurrence. Overall, the amelioration of the off-target specificity in the CRISPR-Cas9 system undoubtedly will deliver solid genotype-phenotype associations, and therefore empower faithful interpretation of gene-editing statistics, facilitating the basic and clinical utility of this CRISPR-Cas9 technology [142].

## Conclusion and prospect

The progress of genomic editing techniques enlarges the landscape of CAR-T cell-based therapies for adoptive cell therapy. Among the several technologies that can be exploited, CRISPR/Cas9 is comparatively easy to use, simple to design, and cost-effective concurrently remarkable multiplex genome engineering competencies [143]. Now, CRISPR/Cas9-based genome editing provides the capability of further streamlining immune cell-based therapies, more prominently, through the generation of a universal “off-the-shelf” cellular product or engineering these redirected effector cells to overcome resistance in human malignancies, ranging from hematological malignancies to solid tumors [144]. These findings have resulted in the execution of several clinical trials to evaluate the therapeutic safety and efficacy of CRISPR/Cas9-mediated genome editing in CAR-T cell therapy (Table 4). However, for further human trials, designing and expanding large-scale approaches for CRISPR/Cas9-mediated target ablation in mature T cells is of principal significance. These protocols must simplify the transference of sgRNA, and Cas9 concomitant with a gene encoding the CAR, maintain cell survival and support strong in vitro cultivation of modified T cells upon genetic manipulation [119]. These means may comprise transduction of CRISPR/Cas9 machinery and CAR transgenes employing the retroviruses or lentiviruses [145, 146] or using non-integrating viruses, including adenoviruses and adenovirus-associated viruses (AAV) [147, 148]. Further, the development of innovative strategies to attenuate off-target CRISPR/Cas9 editing, such as varying the Cas9 endonuclease using novel PAM specificities [149], applying the high-fidelity Cas9 variants, and also exploiting truncated sgRNAs can support more prominent consequences in vivo [119]. In sum, we guess that conduction of the more comprehensive studies based on the CRISPR-Cas9 application to improve CAR-T cell safety, efficacy, and accessibility could lead to the desired therapeutic outcomes in the clinic.

**Table 4:** Clinical trials based on the use of CRISPR-Cas9 technology to provide more effective and universal CAR-T cell registered in ClinicalTrials.gov (June 2021)

Condition	CAR	Target locus (knocked out)	Phase	Location	Participant number	NCT number
ALL	CD19	MAP4K1 (HPK1)	1	China	40	NCT04037566
NHL	CD19	NA	1	USA	50	NCT04637763
Solid tumor	Mesothelin	PD-1 TRAC	1	China	10	NCT03545815
BCL	CD19 CD20 CD22	N.A	1/2	China	80	NCT03398967
Solid tumor	Mesothelin	PD-1	1	China	10	NCT03747965
BCL	CD19	TRAC B2M	1/2	China	80	NCT03166878

Note: *ALL* acute lymphoblastic leukemia, *BCL* B cell lymphoma, *TRAC* T cell receptor alpha constant, *B2M* beta-2-microglobulin, *PDCD1 or PD1* programmed cell death protein 1, *NHL* non-Hodgkin lymphoma, *MAP4K1* mitogen-activated protein kinase kinase kinase kinase 1, *NA* not available

## Data Availability

Not applicable

## Abbreviations

CAR: Chimeric antigen receptor
GVHD: Graft-versus-host disease
CRS: Cytokine release syndrome
CRISPR: Clustered regularly interspaced short palindromic repeat
ZFN: Zinc finger nuclease
TALEN: Transcription activator-like effector nuclease
TGF-β: Transforming growth factor beta
TRAC: T cell receptor alpha constant
B2M: Beta-2-microglobulin
PD1: Programmed cell death protein 1
sgRNA: Single-stranded guide RNA
